# Supplementary material for: Synthesis of a Chiral 3,6T22-Zn-MOF with a T-Shaped Bifunctional Pyrazole-Isophthalate Ligand Following the Principles of the Supramolecular Building Layer Approach
Source: Molecules. 2022 Aug 23;27(17):5374. doi: 10.3390/molecules27175374 (PMC9457947; doi:10.3390/molecules27175374)
Supplement: Supplementary file 1 [file molecules-27-05374-s001.zip › molecules-1854000-supplementary.pdf]

Supporting Information

Synthesis of a Chiral 3,6T22-Zn-MOF with a T-Shaped Bifunctional Pyrazole-Isophthalate Ligand Following the Principles of the Supramolecular Building Layer Approach

Dennis Woschko, Simon Millan, Muhammed-Ali Ceyran, Robert Oestreich and Christoph Janiak *

Institut für Anorganische Chemie und Strukturchemie, Heinrich-Heine-Universität Düsseldorf, 40225 Düsseldorf, Germany
* Correspondence: janiak@uni-duesseldorf.de; Tel.: +49-211-81-12286

Synthesis of H_2Isa-az-tmpz

Dimethyl 5-(2-(2,4-dioxopentan-3-ylidene)hydrazineyl)isophthalate

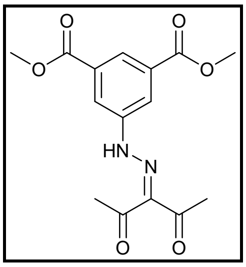

The synthesis of dimethyl 5-(2-(2,4-dioxopentan-3-ylidene)hydrazineyl)isophthalate was carried out following a previously published procedure by Millan et al. Dimethyl 5-aminoisophthalate (2.09 g; 10.0 mmol) was suspended in 20 mL of 3 mol L^{-1} HCL at 0°C. $NaNO_2$ (0.69 g; 10.0 mmol) dissolved in 10 mL of de-ionized (DI) water was slowly added dropwise via a dropping funnel. The diazonium salt solution was then added to a cooled solution of NaOH (0.54 g; 13.5 mmol), NaOAc (4.09 g; 49.9 mmol) and acetylacetone (1.05 mL; 10.0 mmol) in 80 mL of MeOH and 80 mL of DI water, yielding a yellow precipitate. The suspension was stirred for 30 min at 0 °C at first and afterwards for 1 h at room temperature. The yellow precipitate was collected by suction and dried on air and after which the product was recrystallized from ethanole (200 mL). The resulting fibrous yellow product was collected by suction and dried at 60°C under vacuum (2.89 g; 9.0 mmol, 90 %).

^{1}H-NMR (300 MHz, $CDCl_3$, δ [ppm]): 14.66 (s, 1H, NH), 8.45 (t, J = 1.50 Hz, 1H, Ar H), 8.20 (d, J = 1.50 Hz, 2H, Ar H), 3.96 (s, 6H, $-CH_3$), 2.61 (s, 3H, $-CH_3$), 2.52 (s, 3H, $-CH_3$).
^{13}C-NMR (75 MHz, $CDCl_3$, δ [ppm]): 198.55, 197.10, 165.63, 142.43, 134.23, 132.32, 127.20, 120.94, 52.81, 31.87, 26.82.
EA [%] calc. for $C_{15}H_{16}N_2O_6$, C 56.25, H 5.04, N 8.57; found C 56.49, H 4.92, N 8.69.

ESI-MS (%)**:** $[M+H]^+$ 321.3 (100).

Dimethyl 5-(2-(1,3,5-trimethyl-1H-pyrazol-4-yl)azo)-isophthalate

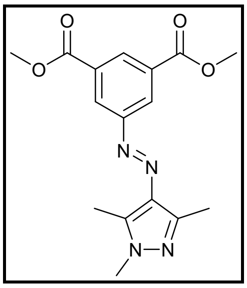

Methylhydrazine (105 µL; 2.0 mmol) was added to a solution of Dimethyl 5-(2-(2,4-dioxopentan-3-ylidene)hydrazineyl)isophthalate (640.6 mg; 2.0 mmol) in 70 mL EtOH and the mixture was refluxed for 6 h. Afterwards the solution was concentrated under reduced pressure and quenched with DI water. The yellow powder was then filtrated and dried in a vacuum oven over night at 60 °C yielding 638.0 mg (1.93 mmol; 96 %) of Dimethyl 5-(2-(1,3,5-trimethyl-1H-pyrazol-4-yl)azo)-isophthalate. The product was used without further purification.

^{1}H-NMR (300 MHz, $CDCl_3$, δ [ppm]): 8.67 (t, J = 1.61 Hz, 1H, Ar H), 8.57 (d, J = 1.71 Hz, 2H, Ar H), 3.98 (s, 6H, $-CH_3$), 3.80 (s, 3H, $-NCH_3$), 2.61 (s, 3H, $-CH_3$), 2.51 (s, 3H, $-CH_3$).
^{13}C-NMR (75 MHz, $CDCl_3$, δ [ppm]): 166.23, 153.93, 142.77, 140.18, 135.39, 131.60, 130.81, 126.98, 52.66, 36.19, 14.12, 10.21.
EA [%] calc. for $C_{16}H_{18}N_4O_4$, C 58.17, H 5.49, N 16.96; found C 58.43, H 5.49, N 16.76.
ESI-MS (%)**:** $[M+H]^+$ 331.3 (100).

5-(2-(1,3,5-trimethyl-1H-pyrazol-4-yl)azo)isophthalic acid (H_2Isa-az-tmpz)

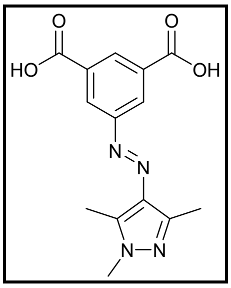

Dimethyl 5-(2-(1,3,5-trimethyl-1H-pyrazol-4-yl)azo)-isophthalate (600 mg; 1.82 mmol) was dissolved in 35 mL MeOH, 10 mL of DI-water and 4.08 g (72.8 mmol) of KOH and refluxed for 24 h. MeOH was removed under reduced pressure and the pH of the remaining yellow solution was adjusted to pH 3 with HCL (3 mol L^{-1}). The yellow precipitate was filtrated, washed with DI water and dried at 60 °C in a vacuum oven to yield 485.0 mg (1.60 mmol; 88 %) of H_2Isa-az-tmpz.

^{1}H-NMR (300 MHz, DMSO-d6, δ [ppm]): 8.45 (t, J = 1.61 Hz, 1H, Ar H), 8.36 (d, J = 1.62 Hz, 2H, Ar H), 3.74 (s, 3H, $-NCH_3$), 2.55 (s, 3H, $-CH_3$), 2.36 (s, 3H, $-CH_3$).
^{13}C-NMR (75 MHz, DMSO-d6, δ [ppm]): 166.26, 153.10, 140.85, 140.51, 134.55, 132.37, 130.03, 125.58, 36.00, 13.84, 9.58.
EA [%] calc. for $C_{14}H_{14}N_4O_4$, C 55.63, H 4.67, N 18.53; found C 55.66, H 4.71, N 18.16.
EI-MS (%)**:** $[M]^+$ 302 (36), $[Az\text{-}tmpz]^+$ 137 (100), $[Tmpz]^+$ 109 (55).

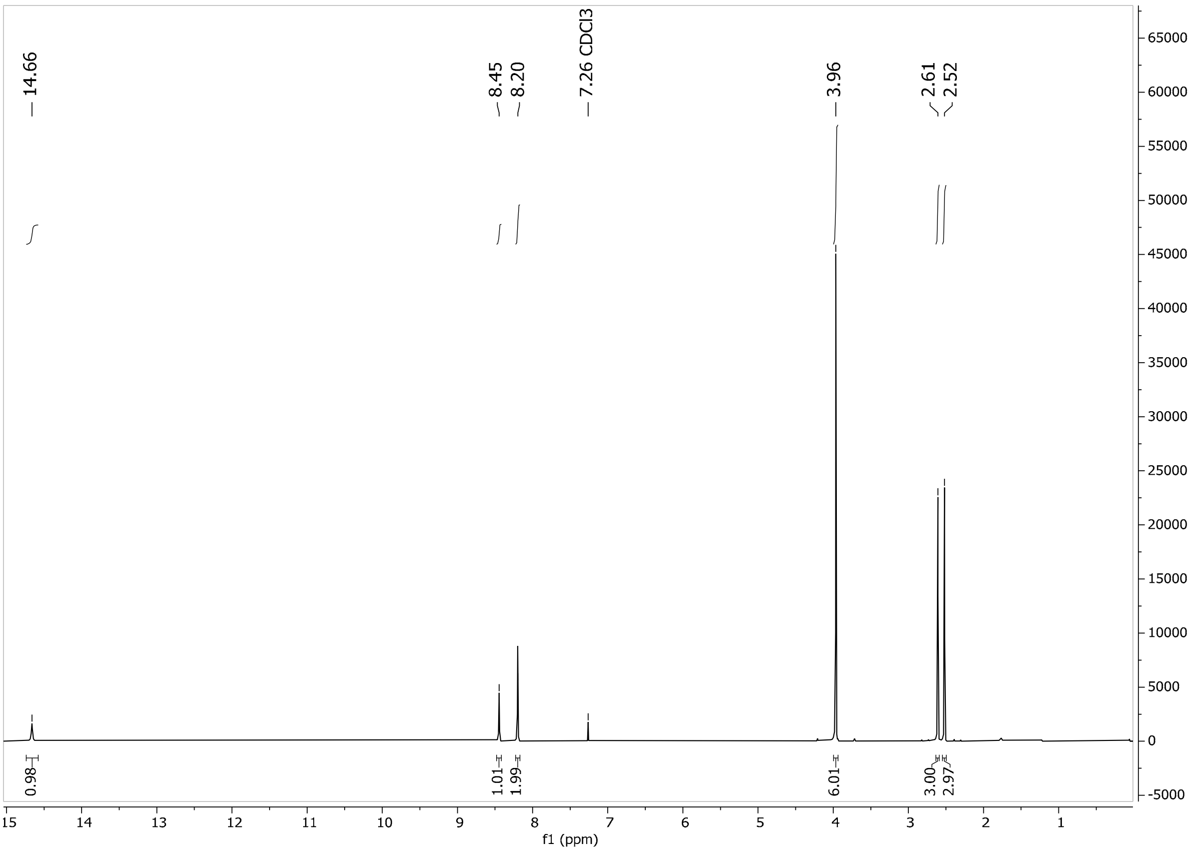

Figure S1. ^{1}H-NMR spectrum of dimethyl 5-(2-(2,4-dioxopentan-3-ylidene)hydrazineyl)-isophthalate in $CDCl_3$.

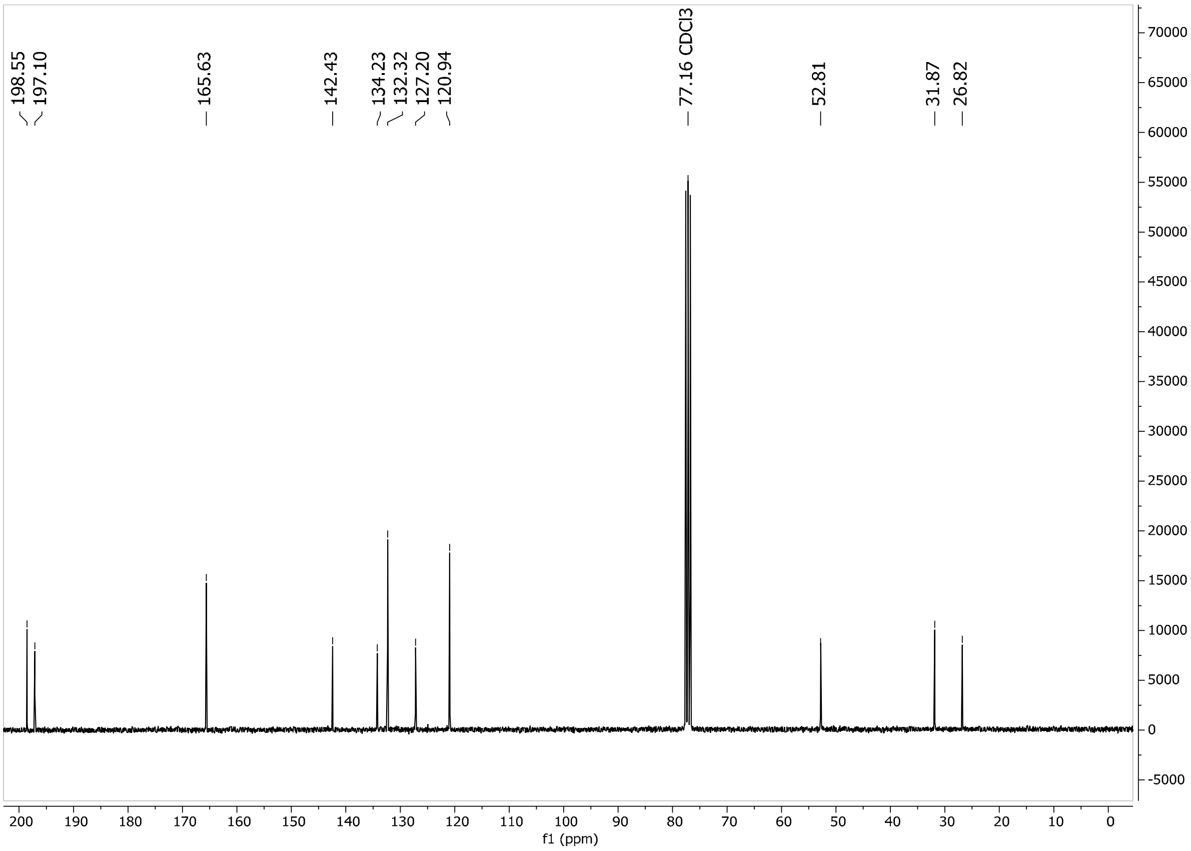

Figure S2. ^{13}C-NMR spectrum of dimethyl 5-(2-(2,4-dioxopentan-3-ylidene)hydrazineyl)-isophthalate in $CDCl_3$.

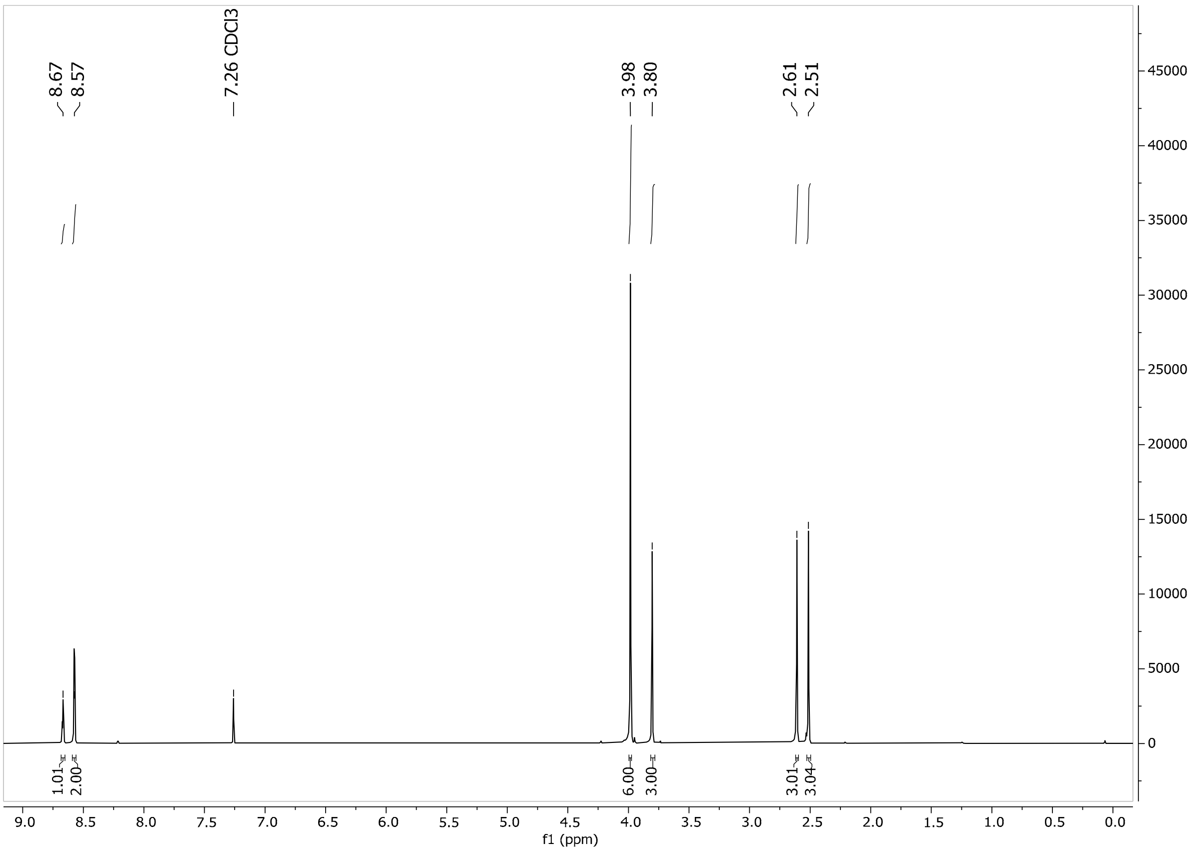

Figure S3. ¹H-NMR spectrum of dimethyl 5-(2-(1,3,5-trimethyl-1H-pyrazol-4-yl)azo)-isophthalate in $CDCl_3$.

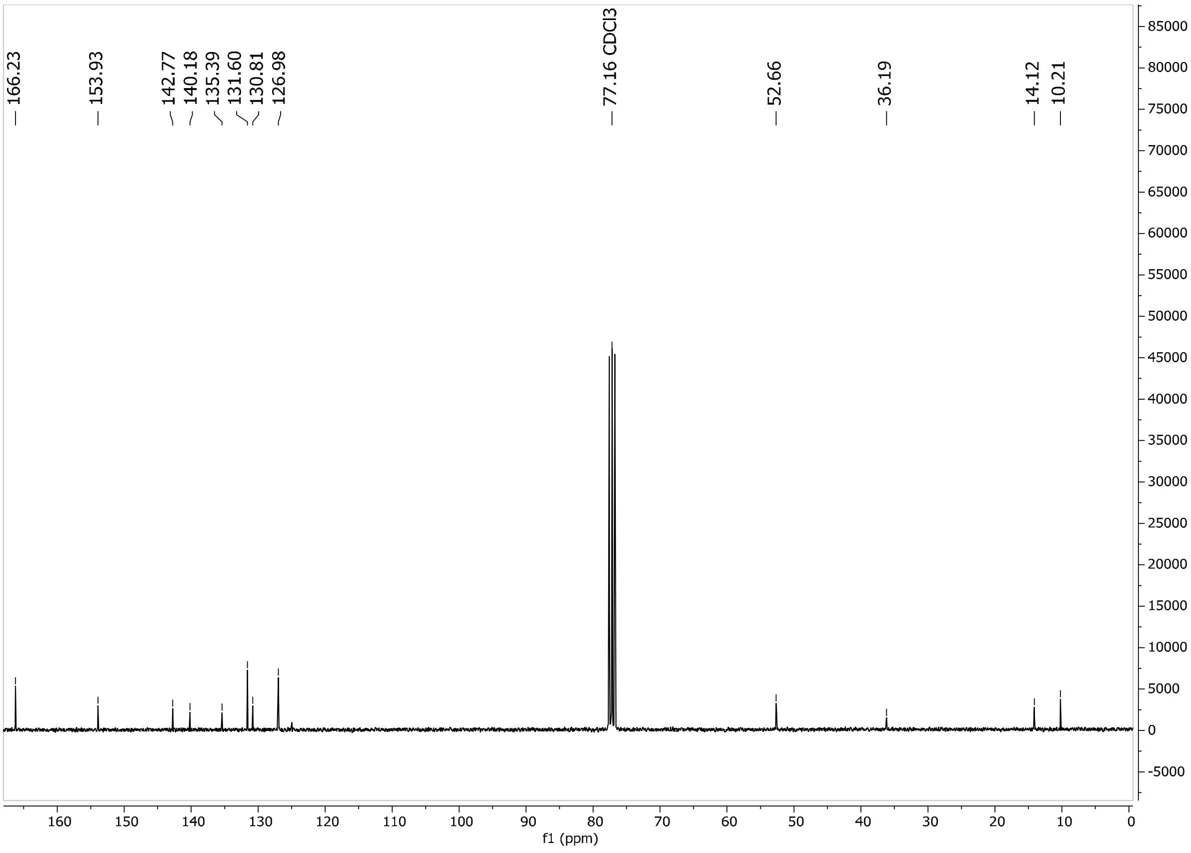

Figure S4. ¹³C-NMR spectrum of dimethyl 5-(2-(1,3,5-trimethyl-1H-pyrazol-4-yl)azo)-isophthalate in $CDCl_3$.

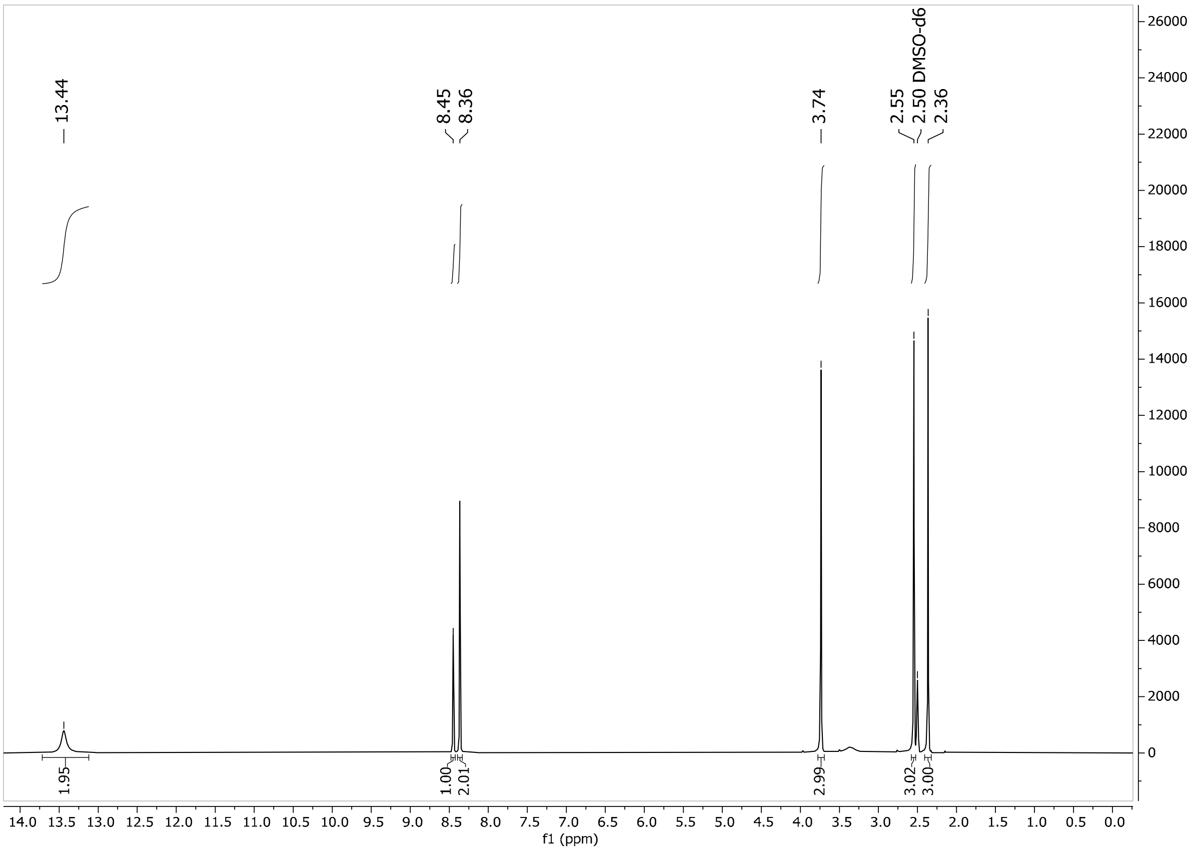

Figure S5. ^{1}H-NMR spectrum of H_2Isa-az-tmpz in DMSO-d6.

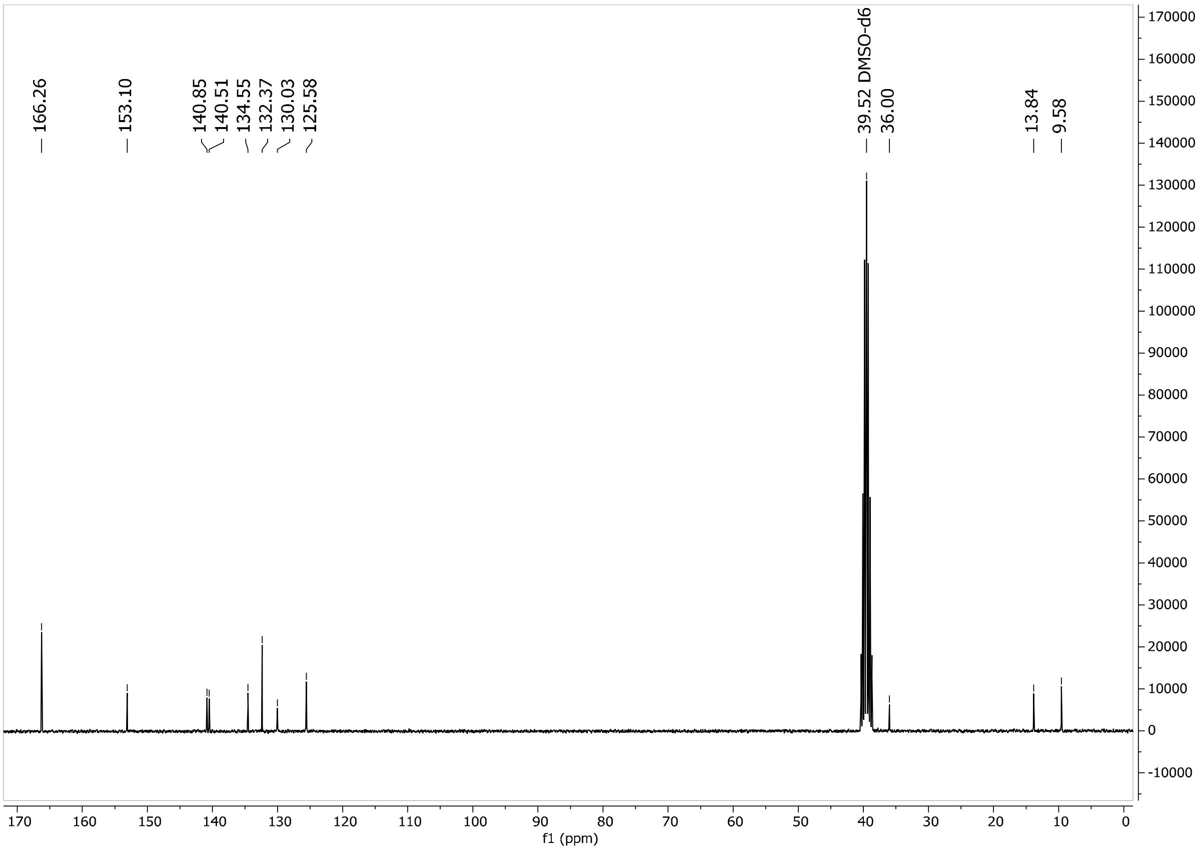

Figure S6. ^{13}C-NMR spectrum of H_2Isa-az-tmpz in DMSO-d6.

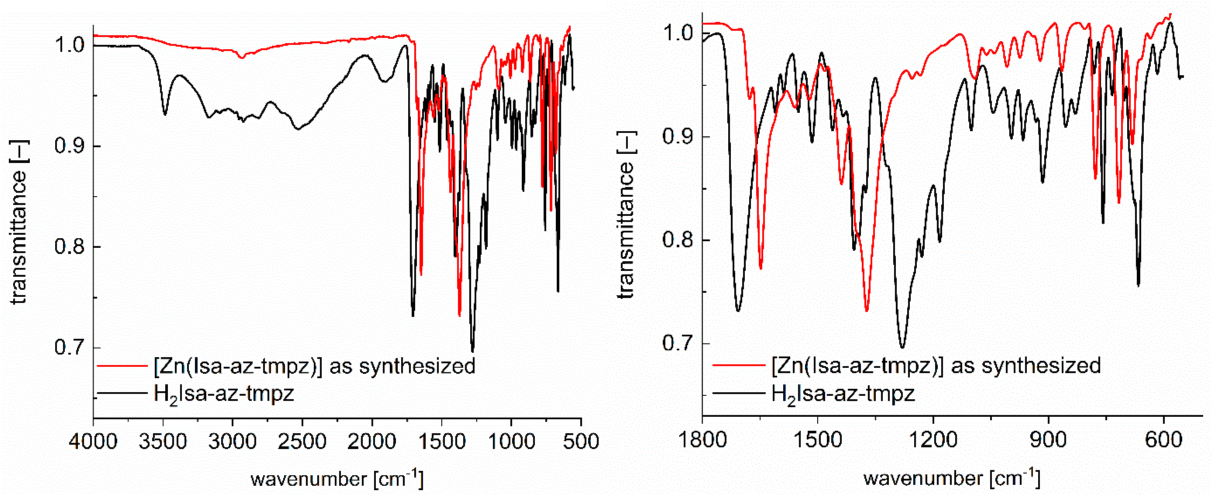

Figure S7. IR-spectra of [Zn(Isa-az-tmpz)]·~1–1.5 DMF and H_2Isa-az-tmpz with full range (left) and enlarged section from 1800 to 500 cm^{-1} (right).

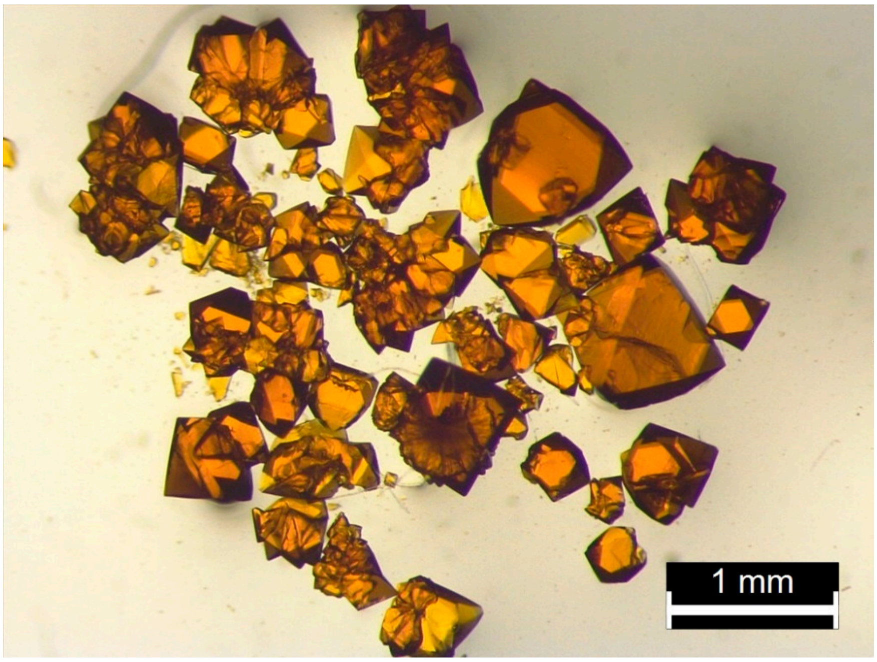

Figure S8. Crystals of [Zn(Isa-az-tmpz)]·~1–1.5 DMF obtained from a single crystal synthesis.

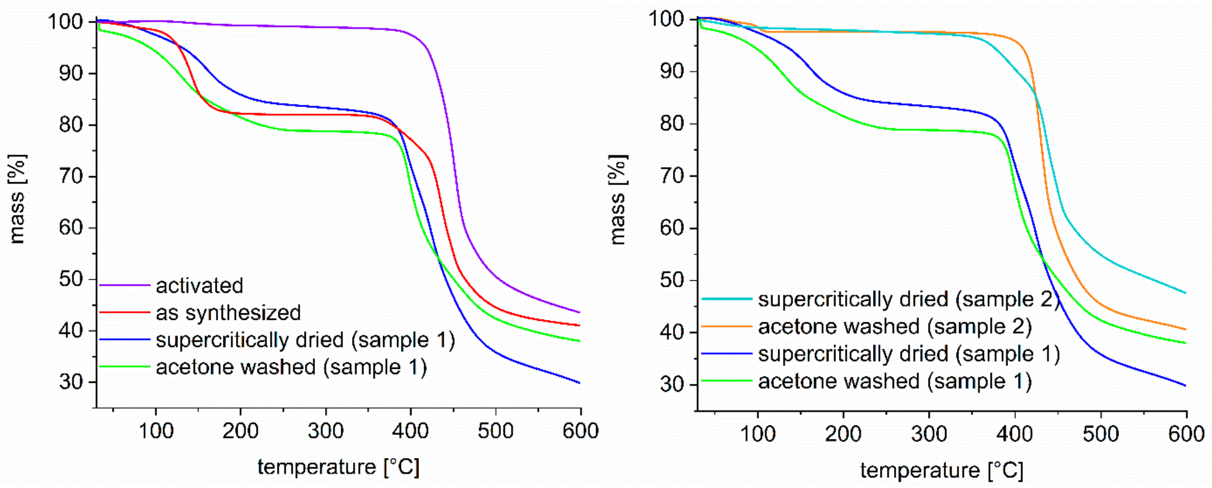

Figure S9. Thermogravimetric analysis (TGA) of [Zn(isa-az-tmpz)] after sequential activation steps. The “activated” material was derived from the supercritically dried sample through additional heating for 3 h at 120 °C under high vacuum.

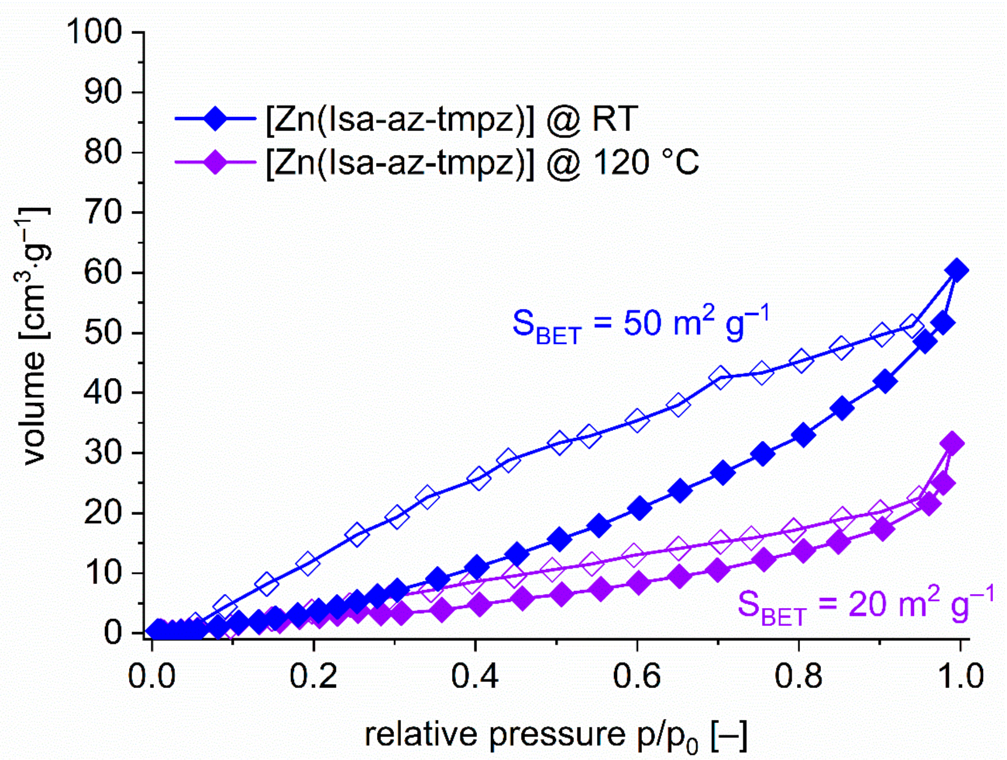

Figure S10. Volumetric nitrogen sorption measurements of [Zn(isa-az-tmpz)] activated at room temperature (RT) and 120 °C.

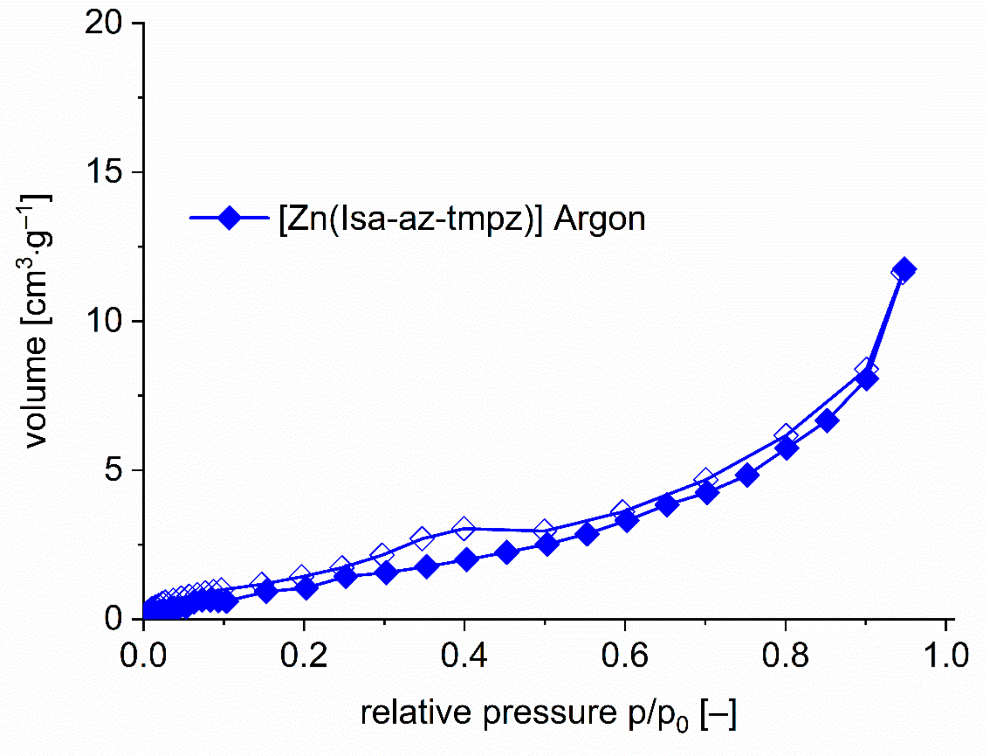

Figure S11. Volumetric argon sorption measurement of [Zn(isa-az-tmpz)] activated at 120 °C.

Table S1. Theoretical surface area and pore volume of [Zn(isa-az-tmpz)]. Calculations are based on structural data obtained from Crystal 1a.

**Mercury 'void' calculation**[1]	
Probe radius 1.2 Å, grid spacing 0.7 Å<br>Void volume [Å^3] (% of unit cell)<br>specific [cm^3 g^{-1}]	 1406 (33)<br>0.29
Probe radius 0.7 Å, grid spacing 0.7 Å<br>Void volume [Å^3] (% of unit cell)<br>specific [cm^3 g^{-1}]	 1693 (40)<br>0.35
**Platon 'Calc Void'**[2,3]	
Total potential solvent area [Å^3] (% of unit cell)<br>specific [cm^3 g^{-1}]	1661 (39)<br>0.34
**CrystalExplorer 'Crystal voids' calculation**[4,5]	
Surface area $S_{unit\ cell}$ (isovalue 0.002) [Å^2]<br>specific [m^2 g^{-1}]	1672<br>3442
Surface area $S_{unit\ cell}$ (isovalue 0.003) [Å^2]<br>specific [m^2 g^{-1}]	1747<br>3596
Pore volume (isovalue 0.002) [Å^2]<br>specific [cm^3 g^{-1}]	1753<br>0.36
Pore volume (isovalue 0.003) [Å^2]<br>specific [cm^3 g^{-1}]	1983<br>0.41

Theoretical specific surface areas are calculated according to ($S_{Unit\ Cell} \cdot N_A$)/(Z · M) and theoretical specific pore volumes are calculated according to (Void Volume · N_A)/(Z · M) or (Solvent Accessible Volume · N_A)/(Z · M);

Z = 8 (number of asymmetric formula units), N_A = Avogadro's constant: $6.022 \cdot 10^{23}$ mol^{-1}, M = 365.65 g mol^{-1} (molecular weight of the asymmetric formula unit).

Topology analysis for [Zn(isa-az-tmpz)] with ToposPro.[6,7] The analysis is based on the structural data obtained from Crystal 1a.

```
###################################################
4:C14 H12 N4 O4 Zn/intercluster bonds for rings>6
###################################################


Topology for ZA1
--------------------
Atom ZA1 links by bridge ligands and has
Common vertex with                        R(A-A)
ZB 1   0.1726  -0.1726   0.7500   (-1 0 1)    6.740A     1
ZB 1   0.6726   0.6726   0.5000   ( 0 0 0)    7.556A     1
ZB 1   0.1726   0.8274   0.7500   (-1 1 1)    8.118A     1
Topology for ZB1
--------------------
Atom ZB1 links by bridge ligands and has
Common vertex with                        R(A-A)
ZA 1   0.9026   0.2342   0.5854   ( 0 0 1)    6.740A     1
ZA 1   0.2342   0.9026   0.4146   ( 0 0-1)    6.740A     1
ZA 1   0.4026   0.2658   0.6646   ( 0 0 0)    7.556A     1
ZA 1   0.2658   0.4026   0.3354   ( 0 0 1)    7.556A     1
ZA 1   0.9026   1.2342   0.5854   ( 0 1 1)    8.118A     1
ZA 1   1.2342   0.9026   0.4146   ( 1 0-1)    8.118A     1
-------------------------
Structural group analysis
-------------------------


-------------------------
Structural group No 1
-------------------------
Structure consists of 3D framework with ZBZA2


Coordination sequences
----------------------
ZA1:  1  2  3   4   5   6   7   8   9   10
```

Num   3 15 22 71 61 167 120 303 199 481

Cum   4 19 41 112 173 340 460 763 962 1443

ZB1: 1 2 3   4   5   6   7   8   9   10

Num   6 12 44 42 122 92 240 162 398 252

Cum   7 19 63 105 227 319 559 721 1119 1371

TD10=1419

Vertex symbols for selected sublattice

ZA1 Point symbol:{6^3}

Extended point symbol:[6(3).6(3).6(3)]

Rings coincide with circuits

ZB1 Point symbol:{6^11.8^4}

Extended point symbol:[6.6.6.6.6(2).6(2).6(2).6(2).6(2).6(2).6(2).8(5).8(5).8(3).8(3)]

Vertex symbol: [6.6.6.6.6(2).6(2).6(2).6(2).6(2).6(2).6(2).8.8.*.*]

ATTENTION! Some rings * are bigger than 10, so likely no rings are contained in that angle

----

Point symbol for net: {6^11.8^4}{6^3}2

3,6-c net with stoichiometry (3-c)2(6-c); 2-nodal net

New topology, please, contact the authors (17828 types in 4 databases)

Elapsed time: 5.99 sec.

Result of further analysis in the Topcryst[8,9] database

Topological type: **3,6T22**

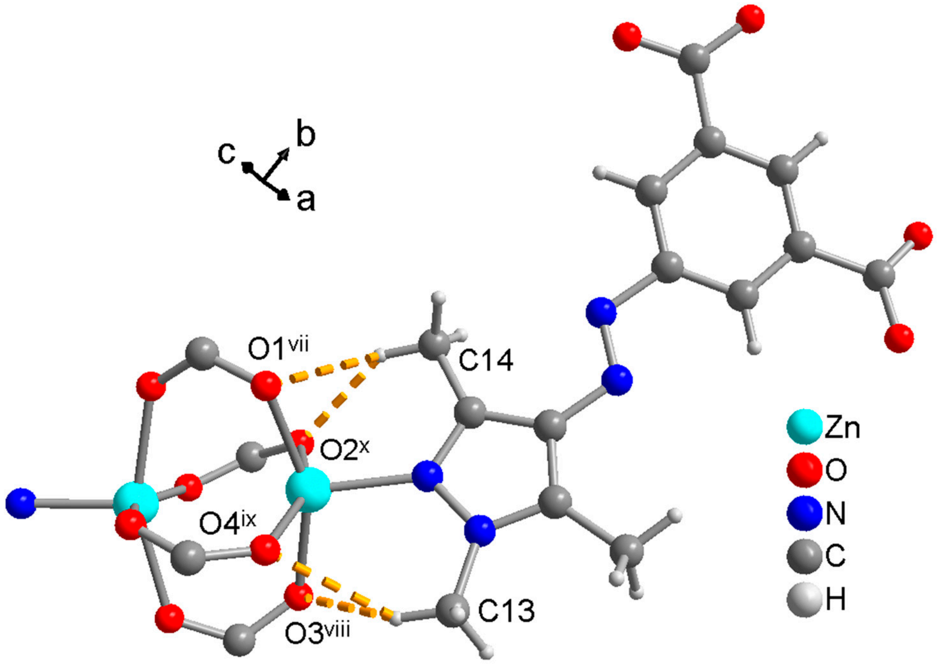

Figure S12. Hydrogen bonds in [Zn(Isa-az-tmpz)]. Symmetry transformations: (vii) y-1/2, -x+1/2, z+1/4; (viii) x, y-1, z; (ix) -y+1, -x, -z+3/2; (x) x-1/2, -y+1/2, -z+5/4. Image created from the structural data of crystal 1a.

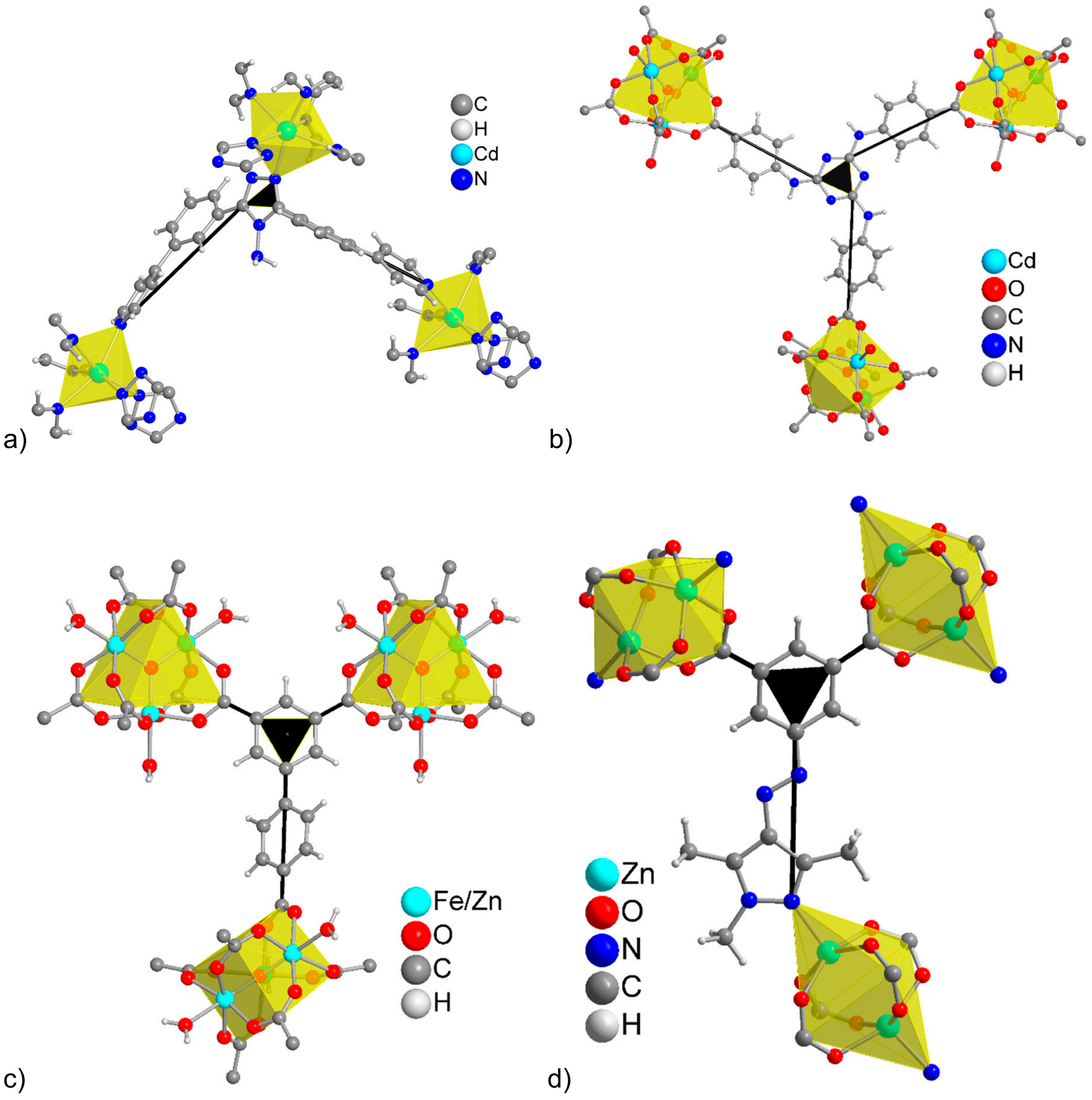

Figure S13. Representations of the metal-linker connectivities in the **3,6T22** MOFs (a) G⊂Cd(L)$_2$ [10,11], (b) [(Me$_2$NH$_2$)Cd$_3$(OH) (H$_2$O)$_3$(Tatab)$_2$] [12], (c) [Fe$_2$M(Bptc)] [13] and (d) [Zn(Isa-az-tmpz)] (this work) The metal SBUs are drawn as yellow 6-c nodes and the linkers are depicted as black 3-c nodes. (L = 4-amino-3,5-bis(4-pyridyl-3-phenyl)-1,2,4-triazole, Tatab = 4,4′,4″-s-triazin-1,3,5-triyltri-p-amino-benzoate, Bptc = biphenyl-3,4′,5-tricarboxylate).

Table S2. Selected bond lengths [Å] and angles [°] for [Zn(isa-az-tmpz)] in *Crystal 1a*.

Zn1—O1	2.0242 (16)	N3—C10	1.338 (3)
Zn1—N4^{i}	2.0387 (18)	N3—N4	1.365 (3)
Zn1—O3^{ii}	2.0459 (15)	N3—C13	1.464 (3)
Zn1—O4^{iii}	2.0575 (15)	O3—C8	1.265 (2)
Zn1—O2^{iv}	2.0586 (15)	C3—C4	1.381 (3)
Zn1—Zn1^{iv}	3.0417 (5)	O4—C8	1.251 (3)
O1—C7	1.268 (3)	N4—C11	1.342 (3)
N1—N2	1.259 (3)	C4—C5	1.400 (3)
N1—C3	1.432 (3)	C5—C6	1.378 (3)
C1—C2	1.394 (3)	C5—C8	1.496 (3)
C1—C6	1.395 (3)	C9—C10	1.382 (3)
C1—C7	1.494 (3)	C9—C11	1.410 (3)
O2—C7	1.254 (3)	C10—C12	1.494 (4)
N2—C9	1.397 (3)	C11—C14	1.479 (3)
C2—C3	1.391 (3)		
O1—Zn1—N4i	100.87 (7)	C4—C3—C2	121.1 (2)
O1—Zn1—O3ii	154.84 (6)	C4—C3—N1	114.3 (2)
N4i—Zn1—O3ii	104.27 (7)	C2—C3—N1	124.6 (2)
O1—Zn1—O4iii	88.19 (7)	C8—O4—Zn1vi	124.02 (14)
N4i—Zn1—O4iii	105.33 (7)	C11—N4—N3	106.07 (18)
O3ii—Zn1—O4iii	84.19 (7)	C11—N4—Zn1vii	127.34 (16)
O1—Zn1—O2iv	89.04 (6)	N3—N4—Zn1vii	126.56 (14)
N4i—Zn1—O2iv	99.61 (7)	C3—C4—C5	120.1 (2)
O3ii—Zn1—O2iv	87.86 (7)	C6—C5—C4	118.9 (2)
O4iii—Zn1—O2iv	154.98 (6)	C6—C5—C8	120.8 (2)
O1—Zn1—Zn1iv	79.35 (4)	C4—C5—C8	120.2 (2)
N4i—Zn1—Zn1iv	175.22 (6)	C5—C6—C1	121.2 (2)
O3ii—Zn1—Zn1iv	75.69 (5)	O2—C7—O1	126.2 (2)
O4iii—Zn1—Zn1iv	79.44 (4)	O2—C7—C1	117.7 (2)
O2iv—Zn1—Zn1iv	75.61 (4)	O1—C7—C1	116.04 (19)
C7—O1—Zn1	123.78 (15)	O4—C8—O3	125.9 (2)
N2—N1—C3	114.2 (2)	O4—C8—C5	117.55 (19)

C2—C1—C6	119.7 (2)	O3—C8—C5	116.5 (2)
C2—C1—C7	120.4 (2)	C10—C9—N2	121.7 (2)
C2—C1—C6	119.7 (2)	C10—C9—C11	106.5 (2)
C2—C1—C7	120.4 (2)	N2—C9—C11	131.8 (2)
C6—C1—C7	119.86 (19)	N3—C10—C9	106.5 (2)
C7—O2—Zn1iv	127.28 (14)	N3—C10—C12	122.5 (2)
N1—N2—C9	113.6 (2)	C9—C10—C12	131.0 (3)
C3—C2—C1	118.9 (2)	N4—C11—C9	109.0 (2)
C10—N3—N4	111.92 (19)	N4—C11—C14	121.7 (2)
C10—N3—C13	126.7 (2)	C9—C11—C14	129.3 (2)
N4—N3—C13	121.31 (19)	C6—C5—C4	118.9 (2)
C8—O3—Zn1v	125.77 (15)		

Symmetry codes: (i) -*y*+1/2, *x*+1/2, *z*-1/4; (ii) -*y*+3/2, *x*+1/2, *z*-1/4; (iii) *x*+1/2, -*y*+3/2, -*z*+5/4; (iv) *y*, *x*, -*z*+1; (v) *y*-1/2, -*x*+3/2, *z*+1/4; (vi) *x*-1/2, -*y*+3/2, -*z*+5/4; (vii) *y*-1/2, -*x*+1/2, *z*+1/4.

Table S3. Hydrogen bond lengths [Å] and angles [°] for [Zn(isa-az-tmpz)] in *Crystal 1a*.

**D—H···A**	**D—H**	**H···A**	**D···A**	**D—H···A**
C13—H13B···O3^{viii}	0.98	2.51	3.370 (3)	146
C13—H13B···O4^{ix}	0.98	2.52	3.244 (3)	130
C14—H14A···O1v^{ii}	0.98	2.60	3.283 (3)	127
C14—H14A···O2^{x}	0.98	2.31	3.151 (3)	144

Symmetry codes: (vii) y-1/2, -x+1/2, z+1/4; (viii) x, y-1, z; (ix) -y+1, -x, -z+3/2; (x) x-1/2, -y+1/2, -z+5/4.

References

1 Macrae, C.F.; Sovago, I.; Cottrell, S.J.; Galek, P.T.A.; McCabe, P.; Pidcock, E.; Platings, M.; Shields, G.P.; Stevens, J.S.; Towler, M.; Wood, P.A. Mercury 4.0: from visualization to analysis, design and prediction. *J. Appl. Crystallogr.* **2020**, *53*, 226–235, doi:10.1107/S1600576719014092

2 Spek, A.L. PLATON – A Multipurpose Crystallographic Tool (Utrecht University, Utrecht, The Netherlands) **2008**; Farrugia, L.J. Windows implementation, Version 270519 (University of Glasgow, Scotland) **2019**.

3 Spek, A.L. Structure validation in chemical crystallography. *Acta Crystallogr. D Biol. Crystallogr.* **2009**, *65*, 148–155, doi:10.1107/S090744490804362X.

4 Turner, M.J.; McKinnon, J.J.; Jayatilaka, D.; Spackman, M.A. Visualisation and characterisation of voids in crystalline materials. *CrystEngComm* **2011**, *13*, 1804–1813, doi:10.1039/C0CE00683A.

5 McKinnon, J.J.; Spackman, M.A.; Mitchell, A.S. Novel tools for visualizing and exploring intermolecular interactions in molecular crystals. *Acta Crystallogr. B* **2004**, *60*, 627–668, doi:10.1107/S0108768104020300.

6 Blatov, V.A.; Shevchenko, A.P.; Proserpio, D.M. Applied Topological Analysis of Crystal Structures with the Program Package ToposPro. *Cryst. Growth Des.* **2014**, *14*, 3576–3586, doi:10.1021/cg500498k.

7 Alexandrov, E.V.; Blatov, V.A.; Kochetkov, A.V.; Proserpio, D.M. Underlying nets in three-periodic coordination polymers: topology, taxonomy and prediction from a computer-aided analysis of the Cambridge Structural Database. *CrystEngComm* **2011**, *13*, 3947, doi:10.1039/c0ce00636j.

8 Alexandrov, E.V.; Shevchenko, A.P.; Blatov, V.A. Topological Databases: Why Do We Need Them for Design of Coordination Polymers? *Cryst. Growth Des.* **2019**, *19*, 2604–2614, doi:10.1021/acs.cgd.8b01721.

9 Blatov, V.A.; Proserpio, D.M. Topological relations between three-periodic nets. II. Binodal nets. *Acta Crystallogr A Found Crystallogr* **2009**, *65*, 202–212, doi:10.1107/S0108767309006096.

10 Liu, Q.-K.; Ma, J.-P.; Dong, Y.-B. Reversible adsorption and complete separation of volatile chlorocarbons based on a Cd(II)-triazole MOF in a single-crystal-to-single-crystal fashion. *Chem. Commun.* **2011**, *47*, 12343–12345, doi:10.1039/c1cc14477d.

11 Liu, Q.-K.; Ma, J.-P.; Dong, Y.-B. Adsorption and separation of reactive aromatic isomers and generation and stabilization of their radicals within cadmium(II)-triazole metal-organic confined space in a single-crystal-to-single-crystal fashion. *J. Am. Chem. Soc.* **2010**, *132*, 7005–7017, doi:10.1021/ja101807c.

12 Liu, B.-H.; Liu, D.-X.; Yang, K.-Q.; Dong, S.-J.; Li, W.; Wang, Y.-J. A new cluster-based metal-organic framework with triazine backbones for selective luminescent detection of mercury(II) ion. *Inorg. Chem. Commun.* **2018**, *90*, 61–64, doi:10.1016/j.inoche.2018.02.008.

13 Wang, X.-L.; Dong, L.-Z.; Qiao, M.; Tang, Y.-J.; Liu, J.; Li, Y.; Li, S.-L.; Su, J.-X.; Lan, Y.-Q. Exploring the Performance Improvement of the Oxygen Evolution Reaction in a Stable Bimetal-Organic Framework System. *Angew. Chem. Int. Ed.* **2018**, *57*, 9660–9664, doi:10.1002/anie.201803587.
